# The Editor's Letter-Box

**Published:** 1915-02-20

**Authors:** 


### THE EDITOR'S LETTER-BOX.

### The American Women's War Hospital.

*To the Editor of* The Hospital.

Sir,—The writer of the article on "The Clinical Value of Poor-Law Infirmaries," in your number of January 23, said that panel doctors are required by the Insurance Act to take notes and records of their diagnosis and treatment and that Poor-Law medical officers would have to be prepared to answer the charge why they had not kept such records properly. I pointed out in my rejoinder on January 30 that panel doctors are *not* required to keep any clinical notes except a bare statement of the diagnosis, that Poor-Law medical officers are required by the L.G.B. to keep records of the patient's history, of their examinations of the patient, of the treatment ordered, and of the course of the disease. I added that, to my personal knowledge, these records were faithfully kept in the majority of the large infirmaries, and Dr. Stewart gave point to my statement by detailing the system followed at the Paddington Infirmary. Faced by the necessity of admitting his error, the writer of the article prefers to adopt the familiar expedient of abusing his critic and reiterating his statement. The value of his criticism may be judged from the fact that he is evidently unaware that the system of note-taking described by Dr. Stewart is that in vogue at most large Poor-Law infirmaries.

The grounds for all his sweeping generalities would appear to be his experience as medical officer at one infirmary and the gas-poisoning case—the latter may even be only a special example of the former. He evidently cannot understand that the note "The woman seems in good health now" may be a perfect summary of the case, and that its value depends upon the honesty of the man who made it. Of course, if he did not examine the blood and other organs of the body, the note is valueless, and in a well-managed infirmary such conduct would soon lead to the medical officer guilty of it being required to resign. An experienced medical superintendent accustomed to work at high pressure and to gauge the character and capacity of his medical officer, soon learns to distinguish between essentials and padding. The word "seems" does not necessarily imply a superficial examination. The phrase is habitually used by many medical men who have a due sense of their fallibility and appreciate the niceties of the English language.

It is characteristic of your correspondent's method of reasoning that he now addresses fresh facts in this case without giving any clue as to their bearing on the value of the infirmary record. He says the pallor, emaciation, debility, and nervousness of the woman *when in court* were striking. He does *not* tell us if the symptoms were present when the woman was in the infirmary. If they were, then the infirmary record was valueless, and I am quite prepared to join him in condemning the medical officer for dereliction of duty. But first I want the evidence that these symptoms were present whilst the woman was in the infirmary.

Now as regards the register of *post-mortem* examinations. In the original article the writer says, "In nearly all the Metropolitan infirmaries even quite recently such a register as the *post-mortem* records did not exist." I pointed out that in practically all the Metropolitan infirmaries a record of *post-mortem* examinations was kept and was filed with the clinical notes. To this he can only reply that he wants them kept in a separate book. A *post-mortem* book was kept in this infirmary from the day it was opened. I abolished it ten years ago and filed the record with the clinical notes for this practical reason: if a separate *post-mortem* book is kept and one wishes to refer subsequently to any case in it, one has to consult two indices and collect two lots of papers, one in the clinical records, the other in the *post-mortem* records; if all the records are filed away together, one has to refer to one index only. This is such an elementary principle in record-keeping that I must apologise to your other readers for setting out in full the reasons for it.—Yours faithfully,

Medical Superintendent.

